# Physical activity and breast cancer survival: results from the Nurses’ Health Studies

**DOI:** 10.1093/jncics/pkac085

**Published:** 2022-12-07

**Authors:** Renée Turzanski Fortner, Kristen D Brantley, Shelley S Tworoger, Rulla M Tamimi, Bernard Rosner, Maryam S Farvid, Michelle D Holmes, Walter C Willett, A Heather Eliassen

**Affiliations:** Department of Research, Cancer Registry of Norway, Oslo, Norway; Division of Cancer Epidemiology, German Cancer Research Center, Heidelberg, Germany; Channing Division of Network Medicine, Brigham and Women’s Hospital and Harvard Medical School, Boston, MA, USA; Department of Epidemiology, Harvard T.H. Chan School of Public Health, Boston, MA, USA; Department of Cancer Epidemiology, Moffitt Cancer Center, Tampa, FL, USA; Department of Epidemiology, Harvard T.H. Chan School of Public Health, Boston, MA, USA; Department of Population Health Sciences, Weill Cornell Medicine, New York, NY, USA; Channing Division of Network Medicine, Brigham and Women’s Hospital and Harvard Medical School, Boston, MA, USA; Department of Epidemiology, Harvard T.H. Chan School of Public Health, Boston, MA, USA; Department of Biostatistics, Harvard T.H. Chan School of Public Health, Boston, MA, USA; Department of Epidemiology, Harvard T.H. Chan School of Public Health, Boston, MA, USA; Brown Dermatology Inc, Providence, RI, USA; Channing Division of Network Medicine, Brigham and Women’s Hospital and Harvard Medical School, Boston, MA, USA; Department of Epidemiology, Harvard T.H. Chan School of Public Health, Boston, MA, USA; Channing Division of Network Medicine, Brigham and Women’s Hospital and Harvard Medical School, Boston, MA, USA; Department of Epidemiology, Harvard T.H. Chan School of Public Health, Boston, MA, USA; Department of Nutrition, Harvard T.H. Chan School of Public Health, Boston, MA, USA; Channing Division of Network Medicine, Brigham and Women’s Hospital and Harvard Medical School, Boston, MA, USA; Department of Epidemiology, Harvard T.H. Chan School of Public Health, Boston, MA, USA

## Abstract

**Background:**

Physical activity is generally associated with better outcomes following diagnosis; however, few studies have evaluated change in pre- to postdiagnosis activity and repeated measures of activity by intensity and type.

**Methods:**

We evaluated physical activity and survival following a breast cancer diagnosis in the Nurses’ Health Study and Nurses’ Health Study II (n = 9308 women, n = 1973 deaths). Physical activity was evaluated as updated cumulative average of metabolic equivalent of task (MET)-h/wk (assigned per activity based on duration and intensity) and change in pre- to postdiagnosis activity. Cox proportional hazards models were used to estimate hazard ratios (HRs) and 95% confidence intervals (CIs).

**Results:**

Higher postdiagnosis activity was inversely associated with breast cancer–specific mortality in categories from ≥9 MET-h/wk (vs <3 MET h/wk, HR_≥9 to <18_ = 0.74 [95% CI = 0.55 to 0.99]; HR_≥27_ = 0.69 [95% CI = 0.50 to 0.95]; *P*_trend_ = .04) and all-cause mortality from ≥3 MET-h/wk (HR_≥3 to <9_ = 0.73 [95% CI = 0.61 to 0.88]; HR_≥27_ = 0.51 [95% CI = 0.41 to 0.63]; *P*_trend_ < .001). Associations were predominantly observed for estrogen receptor–positive tumors and in postmenopausal women. Walking was associated with lower risk of all-cause mortality (≥9 vs <3 MET-h/wk, HR= 0.69 [95% CI = 0.57 to 0.84]) as was strength training. Relative to stable activity pre- to postdiagnosis (±3 MET-h/wk), increases from ≥3 to 9 MET-h/wk were associated with lower all-cause mortality risk (*P*_trend_ < .001). Results were robust to adjustment for prediagnosis physical activity.

**Conclusions:**

Physical activity was associated with lower risk of death following diagnosis. Increased pre- to postdiagnosis activity corresponding to at least 1-3 h/wk of walking was associated with lower risk of death. These results provide further impetus for women to increase their activity after a breast cancer diagnosis, though reverse causation cannot be fully excluded.

Higher levels of recreational physical activity are associated with lower risk of breast cancer (1) and improved survival following diagnosis (2). A 37% lower risk of cancer-related mortality for women with relatively high postdiagnosis recreational activity was observed in a recent meta-analysis (2), in line with our previous study in the Nurses’ Health Study (NHS), which reported 40% lower risk of breast cancer–specific mortality among the most active women (3). However, relatively little is known about the impact of changes in pre- to postdiagnosis activity levels on survival following a breast cancer diagnosis (4-7), and findings from these studies are equivocal. More generally, studies on postdiagnosis activity and survival have largely not evaluated specific activity types including detailed assessments of activities such as walking and strength training, nor have they assessed repeated measures of physical activity after diagnosis (ie, updated over follow-up).

Given that increasing physical activity may represent an accessible intervention for many breast cancer patients, understanding the potential impact of changes in pre- to postdiagnosis activity and associations for specific activity types and intensities is critical to delineating forms of activity that may be relevant for improving survival. The current study provides a comprehensive evaluation of recreational physical activity, overall and by type and intensity, and survival following a breast cancer diagnosis in 2 well-characterized cohorts with up to 3 decades of follow-up.

## Methods

### Study population: the NHS and NHSII

The NHS was initiated in 1976 when 121 700 registered nurses, aged 30-55 years, completed and returned a mailed questionnaire (8,9). The NHSII began in 1989 with 116 429 female registered nurses aged 25-42 years, using the same protocols. Participants in both cohorts have been followed biennially to update information on lifestyle factors and ascertain disease diagnoses. The studies were approved by the institutional review boards at Brigham and Women’s Hospital and Harvard T.H. Chan School of Public Health and participating registries as required.

### Breast cancer case ascertainment

Participants reported disease status on the biennial NHS and NHSII questionnaires. Eligible women reported no prior cancer diagnosis before baseline and were diagnosed with invasive breast cancer through June 2016 (NHS) or June 2017 (NHSII). A total of 13 371 participants diagnosed with breast cancer were identified (NHS, n = 8506; NHSII, n = 4865). A study physician confirmed cases through medical record review. Invasiveness, hormone receptor status, and tumor characteristics were abstracted from medical records; patients provided treatment information (eg, chemotherapy, radiation, surgery) via questionnaire. Vital status was ascertained through June 1, 2016 (NHS) or June 1, 2017 (NHS2), using next-of-kin reports, death certificates, and the National Death Index. Cause of death was assigned according to the International Classification of Diseases version 9 (deaths attributable to breast cancer ICD-9: 174-174.9). Given the high confirmation rate by medical record for breast cancer in this cohort (99%) (10), medical record and participant-confirmed cases were included. Study participants diagnosed with stage I-III breast cancer and at least 1 physical activity report at least 12 months after diagnosis were included (n = 9308; NHS, n = 6068; NHSII, n = 3240).

### Physical activity assessment

Recreational physical activity was queried approximately every 4 years via self-administered questionnaire beginning in 1986 (NHS) and 1989 (NHSII). Physical activity was updated in 1988, 1990 (walking only), 1992, 1994, 1996, 1998, 2000, 2004, 2006, 2008, 2010, 2012, and 2014 (2006, 2010: walking, jogging, and running only) for NHS and in 1991, 1997, 2001, 2005, 2009, and 2013 for NHSII. Participants reported time spent weight training or resistance exercise in 2000, 2004, 2008, 2012, and 2014 (NHS) and 2001, 2005, 2009, and 2013 (NHSII).

Participants reported time spent (categories range: 0 min/wk to ≥11 h/wk) engaging in the following activities: walking or hiking outdoors, jogging (≥10 min/mile), running (<10 min/mile), bicycling, calisthenics/aerobics, aerobic dance/rowing machine, tennis/squash/racquetball, lap swimming, and other aerobic recreation. Participants estimated walking pace (easy, casual [<2 mph]; normal, average [2-2.9 mph]; brisk [3-3.9 mph]; very brisk/striding [≥4 mph]). Time spent weight training or engaged in resistance exercise was collected from 2000 (NHS) and 2001 (NHSII). Energy expenditure was estimated by multiplying metabolic equivalent task (MET) values by reported duration for each reported activity giving MET hours per week (11) (eg, 3-9 MET h/wk corresponds to approximately 1-3 h/wk of walking at a pace of 2.5 mph).

Summary exposures included total recreational physical activity (all activities), walking (walking and hiking), and moderate or vigorous activity (walking ≥3 mph, jogging, or running), strength training (weight training and/or resistance exercise with arm and/or leg weights). Physical activity data were carried forward from the previous questionnaire year only in years when it was not queried (ie, for 2002, activity reported in 2000 was used).

### Covariate assessment

Age at each questionnaire was calculated using date of birth and questionnaire return date. Height was collected on the baseline questionnaire. Further covariates (date of collection) included weight (biennially), menopausal hormone therapy use (biennially), smoking status (biennially), alcohol consumption (every 4 years, NHS: from 1980; NHSII: from 1991), aspirin use (biennially). Body mass index (BMI) was calculated using self-reported weight and height in kilograms per meter^2^.

### Statistical analysis

We examined postdiagnosis physical activity and change in pre- to postdiagnosis activity and breast cancer–specific and all-cause mortality among women diagnosed with breast cancer. Cumulative average postdiagnosis physical activity, beginning with activity assessed at least 12 months after diagnosis, was the evaluated exposure, together with change in pre- to postdiagnosis physical activity (last available prediagnosis activity to first reported measure ≥12 months after diagnosis). Cumulative average was calculated as average activity across follow-up questionnaires and updated with each subsequent physical activity assessment.

Cox proportional hazards models were used with time since diagnosis in months as the timescale for the left-truncated survival model, based on date of receipt of the first physical activity questionnaire received at least 12 months after diagnosis and with follow-up to date of death or end of follow-up. The reference category was less than 3 MET h/wk for analyses of postdiagnosis activity and ±3 MET-h/wk for analyses of change in activity, corresponding to less than approximately 1 hour of walking reported at normal or average pace per week. Activity categories were defined using thresholds previously used in the cohorts; associations in intermediate and extreme categories were of interest toward understanding the levels of activity associated with mortality outcomes. Multivariable models controlled for prediagnosis BMI and hormone therapy use, BMI change from prediagnosis, postdiagnosis alcohol consumption, smoking status, aspirin use, neighborhood socioeconomic status (SES) (12), estrogen receptor (ER) and progesterone receptor (PR) status, treatment with tamoxifen, aromatase inhibitor, Herceptin, and/or chemotherapy, and stage at diagnosis (I-III). We controlled for prediagnosis physical activity in an additional model. Further, we evaluated physical activity by individual intensity or type (ie, moderate and vigorous, strength training, other recreational excluding moderate and vigorous and strength training) and adjusted these models for the other activity types (eg, associations for moderate and vigorous activity adjusted for other recreational and strength training activity).

#### Subgroup and sensitivity analyses

We estimated associations stratified by ER and PR status, stage at diagnosis, menopausal status (premenopausal, postmenopausal), and BMI (<25, ≥25 kg/m^2^), and we evaluated heterogeneity in associations by comparing models with and without an interaction term using the likelihood ratio test (LRT). In a sensitivity analysis, cumulative average physical activity was carried forward from the prior questionnaire cycle introducing a lagged exposure update to minimize the impact of decreases in activity because of recurrence or illness proximate to death (ie, reverse causation). Given activity data were collected approximately every 4 years; this resulted in a lag of 2-6 years. In the analysis on change in pre- to postdiagnosis activity, which used the first postdiagnosis physical activity measure at least 12 months after diagnosis, we excluded deaths within 4 years of diagnosis to reduce the impact of reverse causation.

*P* values were considered statistically significant at less than .05; all statistical tests are 2-sided. Analyses were conducted in SAS 9.4 (Cary, NC, USA).

## Results

A total of 9308 women reported physical activity at least once ≥12 months following a breast cancer diagnosis. Mean age at diagnosis was 63 (NHS) and 48 (NHSII) years and the mean time elapsed between diagnosis and first reported physical activity was 26.5 months (mean time between last reported prediagnosis activity and diagnosis = 18.8 months). Women who were more active at the first questionnaire at least 12 months postdiagnosis had lower prevalence of smoking, higher neighborhood SES, and lower BMI than those who were least active (eg, in the NHS ≥27 vs <3 MET h/wk; current smokers: 10% vs 17%; BMI: 25.2 vs 27.7 kg/m^2^; SES: 25% vs 17% highest quintile) (Table 1).

**Table 1.** Characteristics at first questionnaire at least 12 months after diagnosis, among NHS and NHSII participants diagnosed with breast cancer^a^

Characteristics	First physical activity (MET-h/wk) ≥12 months after dx^b^				
	<3	3 to <9	9 to <18	18 to <27	≥27
Nurses’ Health Study	1685	1601	1308	772	702
Mean age at diagnosis (SD), y^c^	63.9 (9.0)	62.9 (8.6)	62.7 (8.5)	62.1 (8.2)	63.4 (8.0)
Postmenopausal at diagnosis, %	86	87	87	87	89
Last prediagnosis physical activity, mean (SD), MET-h/wk^b^	5.4 (7.2)	9.1 (8.0)	13.0 (9.0)	17.2 (9.7)	22.0 (11.0)
First postdiagnosis physical activity, mean (SD), MET-h/wk^b^	1.3 (0.90)	5.8 (1.8)	13.1 (2.6)	22.0 (2.3)	33.7 (3.8)
Prediagnosis BMI, mean (SD), kg/m²	27.7 (5.6)	26.7 (5.0)	26.1 (4.4)	25.7 (4.0)	25.2 (3.7)
Prediagnosis hormone therapy use, %	36	40	44	45	41
Smoking status at diagnosis, current, %	17	11	10	8	10
Alcohol use at diagnosis, mean (SD), g/day	5.7 (11.3)	5.5 (9.8)	5.3 (8.3)	6.8 (9.6)	6.8 (9.3)
Neighborhood SES, %					
Quintile 1	21	22	20	18	19
Quintile 2	21	20	21	22	17
Quintile 3	21	20	20	18	20
Quintile 4	20	20	20	18	19
Quintile 5	17	18	19	23	25
Aspirin use at diagnosis, %	62	65	64	64	66
ER positive, %^d^	72	73	73	76	77
PR positive, % ^d^	58	60	57	63	60
TNM stage I, %	60	62	64	64	66
Stage II	30	29	28	28	27
Stage III	10	8	8	8	6
Treatment					
Hormone therapy only, %	40	43	46	43	45
Chemotherapy only, %	11	13	10	8	8
Hormone and chemotherapy, %	24	21	21	23	22
Nurses’ Health Study II, No.	616	666	696	390	872
Mean age at diagnosis, y^c^	48.9 (6.5)	47.7 (6.7)	47.8 (7.0)	48.0 (6.7)	48.3 (6.6)
Postmenopausal at diagnosis, %	29	28	27	28	31
Last prediagnosis physical activity, mean (SD), MET-h/wk^b^	7.6 (12.5)	9.9 (12.0)	15.3 (14.0)	19.3 (14.7)	37.5 (33.1)
First post-dx activity, mean (SD), MET-h/wk^b^	1.2 (0.82)	5.8 (1.6)	13.2 (2.4)	22.1 (2.2)	49.4 (25.0)
Prediagnosis BMI, mean (SD), kg/m²	28.0 (5.9)	26.5 (5.1)	25.8 (4.9)	25.5 (4.2)	25.1 (4.8)
Prediagnosis hormone therapy use, %	12	10	11	12	14
Smoking status at diagnosis, current, %	14	8	9	7	8
Alcohol use at diagnosis, mean (SD), g/day	4.2 (7.9)	4.3 (6.9)	5.0 (8.8)	4.8 (6.8)	5.6 (8.6)
Neighborhood SES, %					
Quintile 1	24	20	22	17	17
Quintile 2	24	23	20	19	18
Quintile 3	20	23	19	20	20
Quintile 4	17	15	18	22	23
Quintile 5	14	18	21	22	22
Aspirin use at diagnosis, %	41	40	39	41	40
ER positive, %^d^	73	71	74	76	77
PR positive, %^d^	66	65	66	71	70
TNM stage I. %	52	55	52	57	61
Stage II	33	36	35	28	32
Stage III	14	10	12	15	8
Treatment					
Hormone therapy only, %	18	17	22	24	25
Chemotherapy only, %	23	21	19	18	16
Hormone and chemotherapy, %	39	41	41	39	38

a Percentages are for categorical variables and are standardized to the age distribution of the study population. Values of polytomous variables may not sum to 100% because of rounding. BMI = body mass index; dx = diagnosis; ER = estrogen receptor; MET = metabolic equivalent task; NHS = Nurses’ Health Study; NHSII = Nurses’ Health Study II; PR = progesterone receptor; SES = socioeconomic status; TNM = tumor-node-metastasis.

b 3-9 MET-h/wk corresponds to an activity level comparable to approximately 1-3 h/wk of walking at a pace of 2.5 mph.

c Value is not age adjusted.

d ER missing: NHS = 10%, NHSII = 6%; PR missing: NHS = 12%, NHSII = 7%; HER2 missing: NHS = 74%, NHSII = 42%.

Postdiagnosis physical activity was associated with lower breast cancer–specific mortality at higher levels (total activity, vs <3 MET-h/wk, HR_≥9 to <18_ = 0.74 [95% CI = 0.55 to 0.99]; HR_≥27_ = 0.69 [95% CI = 0.50 to 0.95]; *P*_trend_ = .04) and all-cause mortality even at relatively low levels (eg, vs <3 MET-h/wk, HR_≥3 to <9_ = 0.73 [95% CI = 0.61 to 0.88];  HR_≥27_ = 0.51 [95% CI = 0.41 to 0.63]; *P*_trend_ < .001) (Table 2). Higher levels of walking were associated with lower risk of all-cause mortality (eg, ≥9 vs <3 MET-h/wk, HR = 0.69 [95% CI = 0.57 to 0.84]) but not statistically significantly associated with breast cancer–specific mortality (≥9 vs <3 MET-h/wk, HR = 0.84 [95% CI = 0.64 to 1.11]; Supplementary Table 1, available online). Adjustment for prediagnosis activity had minimal impact on the associations.

**Table 2.** Association between postdiagnosis overall and moderate and vigorous physical activity and survival following breast cancer diagnosis: NHS (1986-2016) and NHSII (1989-2017)^a^

Activity type and outcome	MET hours of physical activity per week^b^					*P*_trend_
	<3	3 to <9	9 to <18	18 to <27	≥27	
	HR (95% CI)	HR (95% CI)	HR (95% CI)	HR (95% CI)	HR (95% CI)	
Total physical activity						
Breast cancer–specific death (n = 891)						
No./person-years	122/10 012	251/29 982	240/34 483	130/19 605	146/23 363	
Multivariable adjusted	Referent	0.79 (0.59 to 1.06)	0.74 (0.55 to 0.99)	0.68 (0.49 to 0.95)	0.69 (0.50 to 0.95)	.04
Multivariable adjusted + prediagnosis PA^c^	Referent	0.73 (0.54 to 1.00)	0.64 (0.45 to 0.89)	0.56 (0.38 to 0.84)	0.56 (0.38 to 0.84)	.02
Overall death (n = 1973)						
No./person-years	278/10 012	567/29 982	559/34 483	290/19 605	277/23 363	
Multivariable adjusted	Referent	0.73 (0.61 to 0.88)	0.67 (0.55 to 0.80)	0.65 (0.53 to 0.81)	0.51 (0.41 to 0.63)	<.001
Multivariable adjusted + prediagnosis PA^c^	Referent	0.73 (0.60 to 0.88)	0.65 (0.53 to 0.80)	0.62 (0.49 to 0.79)	0.47 (0.36 to 0.61)	<.001
Moderate and vigorous physical activity						
Breast cancer–specific death (n = 866)						
No./person-years	581/71 191	164/26 805	83/13 605	25/3648	11/1933	
Multivariable adjusted	Referent	0.84 (0.67 to 1.05)	0.90 (0.67 to 1.22)	0.88 (0.51 to 1.51)	0.54 (0.23 to 1.24)	.13
Multivariable adjusted + prediagnosis PA^c^	Referent	0.83 (0.66 to 1.04)	0.88 (0.65 to 1.21)	0.85 (0.49 to 1.49)	0.52 (0.22 to 1.22)	.12
Overall death (n = 1891)						
No./person-years	1359/71 191	330/26 805	144/13 605	41/3648	15/1933	
Multivariable adjusted	Referent	0.74 (0.64 to 0.86)	0.69 (0.56 to 0.85)	0.74 (0.50 to 1.09)	0.50 (0.27 to 0.96)	<.001
Multivariable adjusted + prediagnosis PA^c^	Referent	0.76 (0.65 to 0.89)	0.71 (0.57 to 0.88)	0.76 (0.51 to 1.13)	0.52 (0.27 to 0.99)	<.001

a Multivariable models adjusted for estrogen receptor (ER) and progesterone receptor (PR) status, treatment with tamoxifen, aromatase inhibitor, Herceptin, and/or chemotherapy, stage at diagnosis (I-III), prediagnosis hormone therapy use, prediagnosis BMI, BMI change from prediagnosis to current, alcohol consumption, smoking status, aspirin use, and neighborhood SES. BMI = body mass index; CI = confidence interval; HR = hazard ratio; MET = metabolic equivalent task; NHS = Nurses’ Health Study; NHSII = Nurses’ Health Study II; PA = physical activity; SES = socioeconomic status.

b Physical activity reported at least 12 months following diagnosis with cumulative average of reported activity over follow-up. Note: 3-9 MET-h/wk corresponds to an activity level comparable to approximately 1-3 h/wk of walking at pace of 2.5 mph.

c Additionally adjusted for prediagnosis physical activity.

Statistically significant heterogeneity was observed by hormone receptor status (*P*_het_ ≤ .01) for overall death. Inverse associations were observed only among women with ER-positive/PR-positive tumors (≥27 vs <3 MET-h/wk, HR = 0.47 [95% CI = 0.34 to 0.65]; *P*_trend _< .001; ER-negative/PR-negative: HR = 1.16 [95% CI = 0.39 to 3.43; *P*_trend_ = .61) (Table 3). Sample size precluded an evaluation of statistical heterogeneity of associations by hormone receptor status for breast cancer–specific death; however, a similar pattern of association was observed for this outcome. In analyses of total and moderate and vigorous physical activity by stage at diagnosis (I, II, III), statistically significant associations were observed for total activity and all-cause mortality among those diagnosed at lower stage; however, overall, these analyses were limited by small sample size in the subgroups of stage (Supplementary Table 2, available online). We evaluated associations by menopausal status, with inverse associations for total activity only observed in women postmenopausal at diagnosis (all-cause mortality, *P*_het_ ≤ .05; breast cancer–specific, *P*_het_ ≥ .45; Supplementary Table 3, available online) and no heterogeneity observed for moderate and vigorous activity (*P*_het_ ≥ .60). No statistically significant heterogeneity in associations was observed by BMI (*P*_het_ ≥ .06; Supplementary Table 4, available online).

**Table 3.** Association between postdiagnosis total activity and survival following a breast cancer diagnosis, by tumor hormone receptor status: NHS (1986-2016) and NHSII (1989-2017)^a^

Outcome	MET hours of physical activity, per week^b^					*P*_trend_	*P*_het_
	<3	3 to <9	9 to <18	18 to <27	≥27		
	HR (95% CI)	HR (95% CI)	HR (95% CI)	HR (95% CI)	HR (95% CI)		
Breast cancer–specific death							
ER and PR positive (n = 427)							
No./person-years	65/5973	119/16 857	116/20 171	63/11 772	64/13 629		
Multivariable adjusted	Referent	0.88 (0.56 to 1.40)	0.82 (0.52 to 1.29)	0.67 (0.40 to 1.12)	0.57 (0.34 to 0.95)	.01	^c^
Multivariable adjusted + prediagnosis PA^d^	Referent	0.85 (0.51 to 1.39)	0.71 (0.42 to 1.23)	0.58 (0.31 to 1.07)	0.50 (0.26 to 0.96)	.02	^c^
ER and PR negative (n = 177)^e^							
No./person-years	20/1305	47/4512	44/4562	26/2734	39/3284		
Multivariable adjusted	Referent	1.23 (0.35 to 4.31)	0.51 (0.13 to 2.01)	0.77 (0.18 to 3.39)	1.14 (0.26 to 4.94)	.99	
Multivariable adjusted + prediagnosis PA^d^	Referent	1.38 (0.31 to 6.04)	0.56 (0.10 to 3.27)	0.90 (0.13 to 6.25)	1.51 (0.18 to 12.4)	.58	
Overall death							
ER and PR positive (n = 1054)							
No./person-years	155/5973	299/16 857	313/20 171	153/11 772	134/13 629		
Multivariable adjusted	Referent	0.77 (0.59 to 1.02)	0.66 (0.50 to 0.87)	0.62 (0.45 to 0.84)	0.47 (0.34 to 0.65)	<.001	.01
Multivariable adjusted + prediagnosis PA^d^	Referent	0.75 (0.56 to 1.01)	0.59 (0.43 to 0.81)	0.53 (0.37 to 0.77)	0.39 (0.26 to 0.58)	<.001	<.001
ER and PR negative (n = 293)							
No./person-years	33/1305	84/4512	77/4562	47/2743	51/3284		
Multivariable adjusted	Referent	0.98 (0.40 to 2.41)	0.69 (0.26 to 1.81)	1.08 (0.39 to 2.94)	1.16 (0.39 to 3.43)	.61	
Multivariable adjusted + prediagnosis PA^d^	Referent	1.21 (0.45 to 3.24)	0.88 (0.28 to 2.73)	1.46 (0.42 to 5.02)	1.78 (0.43 to 7.35)	.39	

a Multivariable models adjusted for estrogen receptor (ER) and progesterone receptor (PR) status, treatment with tamoxifen, aromatase inhibitor, Herceptin, and/or chemotherapy, stage at diagnosis (I-III), prediagnosis hormone therapy use, prediagnosis BMI, BMI change from prediagnosis to current, alcohol consumption, smoking status, aspirin use, and neighborhood SES. Physical activity reported at least 12 months following diagnosis with cumulative average of reported activity over follow-up. BMI = body mass index; CI = confidence interval; HR = hazard ratio; MET = metabolic equivalent task; NHS = Nurses’ Health Study; NHSII = Nurses’ Health Study II; PA = physical activity; SES = socioeconomic status.

b 3-9 MET-h/wk corresponds to an activity level comparable to approximately 1-3 h/wk of walking at pace of 2.5 mph.

c *P*_het_ not calculated because of convergence issues altering comparability of models.

d Additionally adjusted for pre-diagnosis physical activity.

e Not adjusted for HER2 status because of convergence issues.

In analyses considering pre- to postdiagnosis change in activity, women who increased their activity by ≥3 to 9 MET-h/wk or more had lower risk of breast cancer–specific death after controlling for prediagnosis activity (before adjustment: *P* = .06; after adjustment: *P* = .02; Table 4). Statistically significantly lower risk of all-cause mortality (*P*_trend_ ≤ .01) was observed in each category of increased activity, relative to stable activity (vs within ±3 MET-h/wk; HR for categories of increased activity in MET-h/wk, HR_≥3 to <9_ = 0.70 [95% CI = 0.60 to 0.83];  HR_≥9 to <18_ = 0.76 [95% CI = 0.63 to 0.92]; HR_≥18_ = 0.73 [95% CI = 0.61 to 0.87]). These associations were robust to adjustment for prediagnosis activity. No statistically significant associations were observed for decreases in activity.

**Table 4.** Association between change in physical activity from pre- to postdiagnosis and survival following a breast cancer diagnosis: NHS (1986-2016) and NHSII (1989-2017)^a^

Outcome	Increase or decrease in MET-h/wk from pre- to postdiagnosis^b^							
	Decrease ≥18	Decrease 9 to <18	Decrease 3 to <9	Stable	Increase 3 to <9	Increase 9 to <18	Increase ≥18	
	HR (95% CI)	HR (95% CI)	HR (95% CI)		HR (95% CI)	HR (95% CI)	HR (95% CI)	*P*_trend_
Breast cancer–specific death (n = 1086)								
No./person-years	81/8047	91/10 384	130/15 014	490/51 057	120/15 900	78/11 701	95/15 588	
Multivariable adjusted	1.01 (0.73 to 1.40)	0.83 (0.61 to 1.12)	1.06 (0.82 to 1.37)	Referent	0.70 (0.55 to 0.91)	0.74 (0.54 to 1.01)	0.80 (0.60 to 1.05)	.06
Multivariable adjusted + prediagnosis PA^c^	1.12 (0.76 to 1.65)	0.87 (0.63 to 1.19)	1.08 (0.83 to 1.39)	Referent	0.70 (0.55 to 0.91)	0.75 (0.55 to 1.01)	0.81 (0.61 to 1.07)	.02
Overall death (n = 2785)								
No./person-years	181/8047	275/10 384	362/15 014	1237/51 057	283/15 900	203/11 701	243/15 588	
Multivariable adjusted	0.98 (0.80 to 1.20)	0.90 (0.75 to 1.06)	0.91 (0.78 to 1.06)	Referent	0.70 (0.60 to 0.83)	0.76 (0.63 to 0.92)	0.73 (0.61 to 0.87)	<.001
Multivariable adjusted + prediagnosis PA^c^	1.22 (0.96 to 1.55)	1.00 (0.83 to 1.20)	0.95 (0.81 to 1.10)	Referent	0.71 (0.60 to 0.83)	0.78 (0.64 to 0.94)	0.76 (0.64 to 0.90)	<.001

a Multivariable models adjusted for estrogen receptor (ER) and progesterone receptor (PR) status, treatment with tamoxifen, aromatase inhibitor, Herceptin, and/or chemotherapy, stage at diagnosis (I-III), prediagnosis hormone therapy use, prediagnosis BMI, weight change from prediagnosis to current, alcohol consumption, smoking status, aspirin use, and neighborhood SES. Postdiagnosis physical activity reported at least 12 months following diagnosis. Additional deaths included in this analysis as exclusions were not made when physical activity was missing. BMI = body mass index; CI = confidence interval; HR = hazard ratio; MET = metabolic equivalent task; NHS = Nurses’ Health Study; NHSII = Nurses’ Health Study II; PA = physical activity; SES = socioeconomic status.

b 3-9 MET-h/wk corresponds to an activity level comparable to approximately 1-3 h/wk of walking at pace of 2.5 mph.

c Additionally adjusted for pre-diagnosis physical activity

Strength training was associated with lower all-cause mortality (≥1 vs 0 MET-h/wk, HR = 0.61 [95% CI = 0.39 to 0.97]; *P*_trend_ = .04); associations were similar in magnitude but not statistically significant after adjustment for prediagnosis activity and inverse but not statistically significant for breast cancer–specific mortality (Table 5). Results were similar in postmenopausal cases (Supplementary Table 5, available online; sample size limited the evaluation in premenopausal cases), and no heterogeneity was observed by BMI (*P*_het_ ≥ .79; Supplementary Table 6, available online; only evaluated for overall survival because of sample size).

**Table 5.** Associations between postdiagnosis strength training (arm and leg weightlifting) activity and breast cancer survival: NHS (2000-2016) and NHSII (2001-2017)^a^

Outcome	MET hours of physical activity per week					*P*_trend_
	0	0 to <1.0		≥1.0		
	HR (95% CI)	HR (95% CI)		HR (95% CI)		
Breast cancer–specific death (n = 215)						
No./person-years	140/17 020	50/10 611		25/5167		
Multivariable adjusted	Referent	0.59 (0.33 to 1.05)		0.51 (0.25 to 1.02)		.06
Multivariable adjusted + prediagnosis PA	Referent	0.60 (0.33 to 1.08)		0.49 (0.23 to 1.06)		.07
Overall death (n = 518)						
No./person-years	332/17 020	137/10 611		49/5167		
Multivariable adjusted	Referent	0.89 (0.64 to 1.25)		0.61 (0.39 to 0.97)		.04
Multivariable adjusted + prediagnosis PA	Referent	0.90 (0.64 to 1.27)		0.65 (0.40 to 1.06)		.09

a Multivariable models adjusted for estrogen receptor and progesterone receptor status, treatment with tamoxifen, aromatase inhibitor, Herceptin, and/or chemotherapy, stage at diagnosis (I-III), prediagnosis hormone therapy use, prediagnosis BMI, BMI change from prediagnosis to current, alcohol consumption, smoking status, aspirin use, and neighborhood SES. Physical activity reported at least 12 months following diagnosis with cumulative average of reported activity over follow-up. BMI = body mass index; CI = confidence interval; HR = hazard ratio; MET = metabolic equivalent task; NHS = Nurses’ Health Study; NHSII = Nurses’ Health Study II; PA = physical activity; SES = socioeconomic status.

We evaluated the individual components of total physical activity (moderate and vigorous, strength training, other; Supplementary Table 7, available online; moderate and vigorous, other activity; Supplementary Table 8, available online) and mutually adjusted for the other activity type(s). Other (non-moderate or vigorous) activity was consistently inversely associated with all-cause mortality.

Finally, we conducted a sensitivity analysis evaluating cumulative average total and moderate and vigorous activity with a lagged update of exposure. Associations for all-cause mortality remained statistically significantly inverse (≥27 vs <3 MET-h/wk, HR = 0.56 [95% CI = 0.46 to 0.68]; *P*_trend_ < .001), whereas those for breast cancer–specific mortality were attenuated and not statistically significant (≥27 vs <3 MET-h/wk, HR = 0.78 [95% CI = 0.57 to 1.07]; *P*_trend_ = .20; Supplementary Table 9, available online). The sensitivity analysis for pre- to postdiagnosis change in activity excluding deaths within 4 years diagnosis yielded results similar to those in the main analysis, with statistically significant inverse associations for all-cause mortality (Supplementary Table 10, available online).

## Discussion

Physical activity was inversely associated with mortality following a breast cancer diagnosis, particularly for the predominant hormone receptor–positive subtype and among postmenopausal women, in this well-characterized population, providing further evidence supporting physical activity in tertiary prevention in breast cancer. Notably, inverse associations between postdiagnosis activity, assessed using repeated questionnaires over follow-up, and risk of death following a breast cancer diagnosis were consistently observed for modest levels of activity. The observed associations for women reporting 3-9 MET-h/wk of activity following diagnosis correspond to an activity level comparable to approximately 1 to 3 hours of walking per week. Further, we controlled for prediagnosis BMI and change in BMI in our analysis and observed no statistically significant heterogeneity in subgroups of BMI, suggesting that these association are not fully explained by body composition.

Higher postdiagnosis activity was associated with lower risk of breast cancer–specific mortality in pre- and postmenopausal women in a recent meta-analysis (high vs low, premenopausal, relative risk [RR] = 0.65 [95% CI = 0.47 to 0.89]; postmenopausal, RR = 0.68 [95% CI = 0.55 to 0.84]) (2). In a prior analysis in the NHS (n = 2987), a 40% lower risk of breast cancer–specific death was observed with higher postdiagnosis physical activity (≥24 vs <3 MET-h/wk, RR = 0.60 [95% CI = 0.40 to 0.89]; *P*_trend_ = .004) (3). These prior studies predominantly included only 1 postdiagnosis measure of activity, whereas we evaluated updated activity over time in the current study, providing an estimate of physical activity accounting for increases or decreases in activity over follow-up. We observed statistically significantly lower risk of breast cancer–specific death with higher levels of total activity (≥9 to 18 MET-h/wk) and lower risk of overall death even with modest levels of at least 3-9 MET hours (eg, ≥1 to 3 hours of walking at pace of 2.5 mph) per week. We observed statistically significant associations with higher reported walking and all-cause mortality, and in detailed analyses delineating between the activity types, activities classified as not moderate or vigorous were consistently associated with lower risk of death, suggesting that activities at the level of jogging or running may not be necessary to achieve a benefit for survival.

We add to the sparse literature on change in pre- to postdiagnosis activity, observing lower risk of death with modest increases in activity after diagnosis (ie, from 3-9 MET-h/wk). Prior studies have shown a benefit of increasing activity after diagnosis (4,6), though findings are not consistent (5,7). Beyond any potential survival benefit associated with increases in postdiagnosis activity, physical activity interventions have been associated with improvements in health-related quality of life and physical and psychosocial functioning in patients (13), underscoring more general benefits of increasing activity. In contrast to the associations observed with increases in activity, we observed no association between decreases in pre- to postdiagnosis activity and survival. One explanation is that women had to have reported higher prediagnosis activity, which is associated with better outcomes after diagnosis (2), to be eligible to have higher levels of decreased activity after diagnosis. Although we cannot fully exclude this explanation, our results were robust to statistical adjustment for prediagnosis activity levels.

Heterogeneity by hormone receptor status has largely not been observed in prior studies of physical activity and survival (14), though overall associations are more consistently observed for hormone responsive disease. Potential biologic mechanisms linking physical activity to survival following a breast cancer diagnosis include alterations in insulin sensitivity, inflammation, adipokines, and immune response, which may be associated with hormone receptor–positive and hormone receptor–negative disease, and sex steroid hormone metabolism, which may differentially impact survival by tumor hormone receptor status (15). We observed statistically significant heterogeneity when examining overall physical activity by hormone receptor status, with statistically significant associations only observed for ER-positive and PR-positive disease. Physical activity–associated changes in hormone metabolism are a likely explanation for these associations, with physical activity shown to decrease circulating estradiol levels (16).

Strength training was associated with lower risk of all-cause mortality after diagnosis. The benefits of this type of activity for physical function and quality of life in breast cancer patients have been shown (17,18). To our knowledge, these activity types have not previously been investigated with respect to survival in breast cancer patients. Based on the data available and limited participation in these activities, we were only able to evaluate strength training based on a measure of arm and leg weight training. An additional limitation is the relatively low levels of strength training activity reported in this study. Further studies should consider further components of strength training in greater detail and across a broader range of exposure with respect to survival.

Our study has strengths and limitations. The biennial follow-up enabled us to update physical activity and covariate data throughout the study period. Although we used self-reported activity to quantify exposure, this approach has demonstrated reliability and validity (19) and has been associated with other outcomes [ie, diabetes (20), cardiovascular disease (21), colon cancer (22), stroke (23)]. In our analyses of survival, we evaluated physical activity reported at least 12 months postdiagnosis to minimize the impact of treatment on activity levels and used a cumulative average exposure measure. Further, in a sensitivity analysis, we used a more conservative approach with lagged update of physical activity to minimize reverse causation and observed inverse, though attenuated, associations. Reverse causation cannot fully be excluded. Finally, NHS and NHSII study participants are predominantly White women (9), and the limited racial or ethnic diversity may limit the generalization of the findings. Further research in study populations with racial and ethnic diversity is needed.

Our results provide further support for the beneficial effect of physical activity on survival following a breast cancer diagnosis, in particular for hormone responsive tumors and in postmenopausal women. Associations were observed with modest levels of activity and for walking. A key finding was that even moderate increases in activity from pre- to postdiagnosis (eg, from 1 to 3 hours of walking per week) were associated with lower risk of death following a breast cancer diagnosis. These findings provide further support for recreational physical activity as an accessible, modifiable exposure associated with improved survival following a breast cancer diagnosis and support for the benefit of increases in activity after diagnosis in these women.

## Supplementary Material

pkac085_Supplementary_Data

## Data Availability

Data used in this study are available by application at nurseshealthstudy.org.

## Notes

**Role of the funder:** The funding sources had no role in the design of the study; collection, analysis, and interpretation of the data; preparation of the manuscript or decision to submit it for publication.

**Author disclosures:** The authors have no disclosures.

**Author contributions:** Conceptualization: RTF, AHE, RMT, WCW, MDH. Formal analysis: KDB. Investigation: AHE, WCW, RMT, BAR. Funding acquisition: AHE, RMT, WCW. Writing—original draft: RTF. Writing—review & editing: RTF, KDB, SST, RMT, BAR, MSF, MDH, WCW, AHE.

**Acknowledgements:** We would like to thank the following state cancer registries for their help: AL, AZ, AR, CA, CO, CT, DE, FL, GA, ID, IL, IN, IA, KY, LA, ME, MD, MA, MI, NE, NH, NJ, NY, NC, ND, OH, OK, OR, PA, RI, SC, TN, TX, VA, WA, WY. The authors assume full responsibility for analyses and interpretation of these data.
