# Supplementary material for: Physical activity and breast cancer survival: results from the Nurses’ Health Studies
Source: JNCI Cancer Spectr. 2022 Dec 7;7(1):pkac085. doi: 10.1093/jncics/pkac085 (PMC9893869; doi:10.1093/jncics/pkac085)
Supplement: pkac085_Supplementary_Data [file pkac085_supplementary_data.pdf]

Supplementary Material

Supplementary Table 1. Association between post-diagnosis walking and survival following a breast cancer diagnosis: NHS (1986-2016) and NHSII (1989-2017)

	MET-hours of walking, per week					
	<3	3- <9		9+		
	HR (95% CI)	HR (95% CI)		HR (95% CI)		p_{trend}
Breast cancer-specific death (n=925)						
n/person-years	638/88991	185/32984		101/19180		
Multivariable Adjusted	ref	0.90	(0.73-1.12)	0.84	(0.64-1.11)	0.18
Multivariable Adjusted + pre-diagnosis PA	ref	0.90	(0.72-1.12)	0.83	(0.62-1.11)	0.18
Overall death (n=1,956)						
n/person-years	1430/88991	349/32984		176/19180		
Multivariable Adjusted	ref	0.74	(0.64-0.86)	0.69	(0.57-0.84)	<0.001
Multivariable Adjusted + pre-diagnosis PA	ref	0.76	(0.65-0.88)	0.71	(0.58-0.87)	<0.001

Multivariable models adjusted for: estrogen receptor (ER)/progesterone receptor (PR) status, treatment with tamoxifen, aromatase inhibitor, Herceptin, and/or chemotherapy, stage at diagnosis (I-III), pre-diagnosis hormone therapy use, pre-diagnosis BMI, BMI change from pre-diagnosis to current, alcohol consumption, smoking status, aspirin use, and neighborhood SES. Physical activity reported at least 12 months following diagnosis with cumulative average of reported activity over follow-up.

Note: 3-9 MET-hours/week corresponds to an activity level comparable to approximately 1-3 hours/week of walking at pace of 2.5 mph

Supplementary Table 2. Association between post-diagnosis overall and moderate/vigorous physical activity and survival following breast cancer diagnosis: NHS (1986-2016) and NHSII (1989-2017) stratified by stage at diagnosis.

	MET-hours of physical activity, per week									
	<3	3- <9		9 - <18		18 - <27		≥27		
	HR (95% CI)	HR (95% CI)		HR (95% CI)		HR (95% CI)		HR (95% CI)		p_{trend}
Total physical activity										
Breast cancer-specific death (n=837)										
Stage I										
n/person-years	24/5357	69/16427		60/19238		35/11170		36/13206		
Multivariable Adjusted	ref	1.34	(0.70-2.57)	0.83	(0.43-1.59)	0.97	(0.48-1.97)	0.72	(0.35-1.47)	0.10
Multivariable Adjusted + pre-diagnosis PA	ref	1.36	(0.69-2.66)	0.79	(0.39-1.62)	0.87	(0.39-1.93)	0.63	(0.27-1.47)	0.10
Stage II										
n/person-years	50/2954		86/9044		96/9974		38/5355		59/6825	
Multivariable Adjusted	ref	0.55	(0.31-1.00)	0.76	(0.42-1.37)	0.55	(0.27-1.09)	0.65	(0.34-1.22)	0.52
Multivariable Adjusted + pre-diagnosis PA	ref	0.59	(0.32-1.10)	0.74	(0.37-1.46)	0.49	(0.22-1.11)	0.53	(0.24-1.18)	0.24
Stage III										
n/person-years	41/933		79/2711		73/3100		47/1666		44/1702	
Multivariable Adjusted	ref	0.20	(0.05-0.81)	0.32	(0.08-1.23)	0.25	(0.06-1.11)	0.32	(0.08-1.30)	0.90
Multivariable Adjusted + pre-diagnosis PA	ref	0.14	(0.03-0.63)	0.20	(0.04-0.91)	0.12	(0.02-0.72)	0.14	(0.02-0.81)	0.27
Overall death (n=1821)										
Stage I	112/5357	250/16427		244/19238		122/11170		109/13206		
n/person-years										
Multivariable Adjusted	ref	0.81	(0.59-1.10)	0.57	(0.41-0.78)	0.63	(0.44-0.90)	0.45	(0.31-0.65)	<0.001
Multivariable Adjusted + pre-diagnosis PA	ref	0.83	(0.59-1.15)	0.55	(0.38-0.80)	0.57	(0.38-0.87)	0.39	(0.25-0.61)	<0.001
Stage II										
n/person-years	97/2954		166/9044		180/9974		86/5355		96/6825	
Multivariable Adjusted	ref	0.57	(0.38-0.88)	0.73	(0.48-1.12)	0.55	(0.34-0.89)	0.58	(0.36-0.94)	0.15
Multivariable Adjusted + pre-diagnosis PA	ref	0.68	(0.43-1.05)	0.89	(0.55-1.42)	0.63	(0.36-1.11)	0.65	(0.37-1.16)	0.31
Stage III										
n/person-years	52/933		105/2711		91/3100		58/1666		53/1702	
Multivariable Adjusted	ref	0.18	(0.05-0.65)	0.35	(0.11-1.17)	0.24	(0.06-0.92)	0.33	(0.09-1.20)	0.99
Multivariable Adjusted + pre-diagnosis PA		0.11	(0.03-0.45)	0.23	(0.06-0.92)	0.12	(0.02-0.62)	0.15	(0.03-0.83)	0.39
Moderate/vigorous physical activity										
Breast cancer-specific death (n=814)										
Stage I										
n/person-years	145/39343	46/15343		17/7380		9/2057		1/1133		
Multivariable Adjusted	ref	0.84	(0.52-1.34)	0.51	(0.25-1.03)	1.27	(0.46-3.54)	0.29	(0.03-2.57)	0.15

Multivariable Adjusted + pre-diagnosis PA	ref	0.82	(0.50-1.33)	0.49	(0.23-1.03)	1.24	(0.43-3.52)	0.29	(0.03-2.66)	0.17
Stage II										
n/person-years	200/20556		65/7639		38/4300		10/1078		3/495	
Multivariable Adjusted	Ref	0.76	(0.49-1.18)	1.13	(0.65-1.96)	1.52	(0.55-4.20)	0.53	(0.10-2.91)	0.82
Multivariable Adjusted + pre-diagnosis PA	Ref	0.75	(0.47-1.20)	1.09	(0.60-1.99)	1.46	(0.52-4.13)	0.46	(0.08-2.58)	0.93
Stage III										
n/person-years	202/6605		42/2088		23/964		6/276		7/159	
Multivariable Adjusted	ref	0.33	(0.13-0.85)	0.34	(0.08-1.47)	0.02	(0.00-0.55)	1.75	(0.18-17.3)	0.10
Multivariable Adjusted + pre-diagnosis PA	ref	0.25	(0.09-0.70)	0.20	(0.04-1.06)	0.02	(0.00-0.46)	1.33	(0.10-17.8)	0.03
Overall death (n=1743)										
Stage I										
n/person-years	581/39343	152/15343		43/7380		18/2057		3/1133		
Multivariable Adjusted	ref	0.75	(0.58-.96)	0.42	(0.28-0.64)	0.83	(0.44-1.54)	0.31	(0.09-1.11)	<0.001
Multivariable Adjusted + pre-diagnosis PA	ref	0.75	(0.58-0.97)	0.42	(0.28-0.65)	0.82	(0.43-1.54)	0.32	(0.09-1.14)	<0.001
Stage II										
n/person-years	403/20556		110/7639		62/4300		15/1078		4/495	
Multivariable Adjusted	ref	0.61	(0.44-0.85)	0.80	(0.52-1.22)	1.53	(0.67-3.47)	0.51	(0.13-2.00)	0.46
Multivariable Adjusted + pre-diagnosis PA	ref	0.62	(0.43-0.88)	0.80	(0.50-1.27)	1.52	(0.65-3.51)	0.42	(0.10-1.77)	0.57
Stage III										
n/person-years	259/6605		49/2088		30/964		7/276		7/159	
Multivariable Adjusted	ref	0.41	(0.18-0.94)	0.43	(0.13-1.40)	0.05	(0.00-0.68)	1.77	(0.19-16.3)	0.10
Multivariable Adjusted + pre-diagnosis PA	ref	0.32	(0.13-0.83)	0.34	(0.09-1.20)	0.04	(0.00-0.58)	1.52	(0.13-17.4)	0.05

Multivariable models adjusted for: estrogen receptor (ER)/progesterone receptor (PR) status, treatment with tamoxifen, aromatase inhibitor, Herceptin, and/or chemotherapy, pre-diagnosis hormone therapy use, pre-diagnosis BMI, BMI change from pre-diagnosis to current, alcohol consumption, smoking status, aspirin use, and neighborhood SES.
Physical activity reported at least 12 months following diagnosis with cumulative average of reported activity over follow-up.

Supplementary Table 3. Association between post-diagnosis overall and moderate/vigorous physical activity and survival among breast cancer patients by menopausal status at diagnosis: NHS (1986-2016) and NHSII (1989-2017)

	MET-hours of physical activity, per week											
	<3	3- <9		9 - <18		18 - <27		≥27				
	HR (95% CI)	HR (95% CI)		HR (95% CI)		HR (95% CI)		HR (95% CI)		p_{trend}	p_{het}
Total physical activity											
Breast cancer-specific death											
Premenopausal at diagnosis (n=274)											
n/person-years	32/2684	73/8616		70/10030		45/6033		54/7599			
Multivariable Adjusted	ref	1.14	(0.55-2.33)	1.14	(0.55-2.38)	1.10	(0.47-2.57)	1.21	(0.54-2.72)	0.76	0.45
Multivariable Adjusted + pre-diagnosis PA	ref	0.89	(0.41-1.94)	0.84	(0.34-2.05)	0.83	(0.30-2.33)	0.96	(0.33-2.81)	0.94	0.50
Postmenopausal at diagnosis (n=596)											
n/person-years	87/7073	175/20661		166/23526		83/13066		85/15038			
Multivariable Adjusted	ref	0.74	(0.53-1.05)	0.71	(0.51-1.00)	0.64	(0.43-0.94)	0.59	(0.40-0.87)	0.01	
Multivariable Adjusted + pre-diagnosis PA	ref	0.68	(0.47-0.98)	0.60	(0.40-0.89)	0.50	(0.31-0.80)	0.45	(0.28-0.73)	0.004	
Overall death											
Premenopausal at diagnosis (n=356)											
n/person-years	37/2684	96/8616		95/10030		58/6033		70/7599			
Multivariable Adjusted	ref	0.97	(0.51-1.85)	1.08	(0.56-2.07)	1.05	(0.51-2.17)	1.01	(0.50-2.04)	0.91	0.03
Multivariable Adjusted + pre-diagnosis PA	ref	0.86	(0.43-1.70)	0.92	(0.43-2.00)	0.93	(0.39-2.19)	0.89	(0.36-2.21)	1.0	0.05
Postmenopausal at diagnosis (n=1,587)											
n/person-years	238/7073	467/20661		455/23526		228/13066		199/15038			
Multivariable Adjusted	ref	0.72	(0.59-0.88)	0.65	(0.53-0.79)	0.64	(0.51-0.80)	0.46	(0.36-0.58)	<0.001	
Multivariable Adjusted + pre-diagnosis PA	ref	0.73	(0.59-0.90)	0.64	(0.51-0.80)	0.60	(0.46-0.79)	0.42	(0.31-0.56)	<0.001	
Moderate/vigorous physical activity											
Breast cancer-specific death											
Premenopausal at diagnosis (n=273)											
n/person-years	169/18814	63/9078		26/4843		9/1383		6/862			
Multivariable Adjusted	ref	0.84	(0.51-1.39)	0.72	(0.36-1.41)	0.84	(0.26-2.80)	0.40	(0.08-1.91)	0.18	0.61
Multivariable Adjusted + pre-diagnosis PA	ref	0.73	(0.43-1.24)	0.62	(0.30-1.29)	0.70	(0.20-2.50)	0.40	(0.08-2.01)	0.13	0.60
Postmenopausal at diagnosis (n=572)											
n/person-years	402/50675	99/16953		54/8325		13/2165		4/967			
Multivariable Adjusted	ref	0.80	(0.61-1.06)	0.99	(0.69-1.41)	0.94	(0.49-1.79)	0.54	(0.18-1.64)	0.39	

Multivariable Adjusted + pre-diagnosis PA	ref	0.79	(0.60-1.06)	0.96	(0.66-1.40)	0.89	(0.46-1.72)	0.52	(0.17-1.59)	0.33	
Overall death											
Premenopausal at diagnosis (n=353)											
n/person-years	220/18814	83/9078		31/4843		12/1383		7/862			
Multivariable Adjusted	ref	1.07	(0.70-1.63)	0.50	(0.28-0.90)	0.88	(0.33-2.30)	0.56	(0.16-1.98)	0.07	0.93
Multivariable Adjusted + pre-diagnosis PA	ref	0.98	(0.63-1.53)	0.44	(0.24-0.82)	0.73	(0.26-2.02)	0.58	(0.15-2.15)	0.05	0.94
Postmenopausal at diagnosis (n=1,508)											
n/person-years	1124/5065	242/16953		110/8325		25/2165		7/967			
Multivariable Adjusted	ref	0.69	(0.58-0.82)	0.74	(0.58-0.93)	0.69	(0.44-1.08)	0.47	(0.21-1.04)	<0.001	
Multivariable Adjusted + pre-diagnosis PA	ref	0.71	(0.63-0.85)	0.77	(0.60-0.98)	0.73	(0.46-1.15)	0.50	(0.22-1.11)	0.002	

Multivariable models adjusted for: estrogen receptor (ER)/progesterone receptor (PR) status, treatment with tamoxifen, aromatase inhibitor, Herceptin, and/or chemotherapy, stage at diagnosis (**I-III**), pre-diagnosis hormone therapy use, pre-diagnosis BMI, BMI change from pre-diagnosis to current, alcohol consumption, smoking status, aspirin use, and neighborhood SES. Physical activity reported at least 12 months following diagnosis with cumulative average of reported activity over follow-up.
Note: 3-9 MET-hours/week corresponds to an activity level comparable to approximately 1-3 hours/week of walking at pace of 2.5 mph

Supplementary Table 4. Associations between post-diagnosis overall and moderate/vigorous physical activity and survival among breast cancer patients by BMI (updated over follow-up): NHS (1986-2016) and NHSII (1989-2017)

	MET-hours of physical activity, per week										
	<3	3- <9		9 - <18		18 - <27		≥27			
	HR (95% CI)	HR (95% CI)		HR (95% CI)		HR (95% CI)		HR (95% CI)		p_{trend}	p_{het}†
Total physical activity											
Breast cancer-specific death											
BMI <25 kg/m²											
n/person-years (n=408)	51/3295	115/11288		120/14946		71/9637		73/13008			
Multivariable Adjusted	ref	0.76	(0.44-1.31)	0.71	(0.41-1.23)	0.56	(0.30-1.03)	0.63	(0.35-1.13)	0.10	0.32
Multivariable Adjusted + pre-diagnosis PA	ref	0.70	(0.39-1.25)	0.60	(0.32-1.14)	0.45	(0.21-0.94)	0.49	(0.24-1.03)	0.08	0.34
BMI ≥25 kg/m² (n=450)											
n/person-years	71/6718	136/18694		120/19537		59/9968		73/10354			
Multivariable Adjusted	ref	0.76	(0.49-1.17)	0.67	(0.44-1.04)	0.86	(0.52-1.44)	0.73	(0.44-1.22)	0.61	
Multivariable Adjusted + pre-diagnosis PA	ref	0.69	(0.44-1.09)	0.58	(0.35-0.95)	0.75	(0.42-1.34)	0.63	(0.34-1.17)	0.51	
Overall death											
BMI <25 kg/m² (n=877)											
n/person-years	120/3295	282/11288		295/14946		164/9637		147/13008			
Multivariable Adjusted	ref	0.83	(0.59-1.16)	0.74	(0.54-1.03)	0.69	(0.47-0.99)	0.47	(0.33-0.68)	<0.001	0.06
Multivariable Adjusted + pre-diagnosis PA	ref	0.87	(0.61-1.24)	0.76	(0.52-1.11)	0.70	(0.45-1.07)	0.46	(0.29-0.72)	<0.001	0.06
BMI ≥25 kg/m² (n=871)											
n/person-years	158/6718	285/18694		264/19537		126/9968		130/10354			
Multivariable Adjusted	ref	0.66	(0.50-0.86)	0.60	(0.45-0.79)	0.69	(0.50-0.94)	0.53	(0.38-0.74)	0.01	
Multivariable Adjusted + pre-diagnosis PA	ref	0.63	(0.47-0.84)	0.55	(0.40-0.75)	0.61	(0.42-0.88)	0.48	(0.32-0.71)	0.02	
Moderate/vigorous physical activity											
Breast cancer-specific death											
BMI <25 kg/m² (n=420)											
n/person-years	258/27121	96/13651		47/7592		11/2352		5/1326			
Multivariable Adjusted	ref	0.88	(0.60-1.30)	0.90	(0.55-1.45)	0.65	(0.26-1.61)	0.16	(0.03-0.84)	0.04	0.13
Multivariable Adjusted + pre-diagnosis PA	ref	0.90	(0.60-1.35)	0.89	(0.52-1.51)	0.63	(0.25-1.60)	0.15	(0.03-0.82)	0.04	0.13
BMI ≥25 kg/m² (n=449)											
n/person-years	325/51086	68/15020		36/6966		14/1556		6/773			
Multivariable Adjusted	ref	0.83	(0.58-1.19)	0.89	(0.53-1.49)	1.34	(0.54-3.30)	1.56	(0.46-5.33)	0.74	

Multivariable Adjusted + pre-diagnosis PA	ref	0.82	(0.57-1.18)	0.86	(0.51-1.46)	1.32	(0.52-3.32)	1.59	(0.45-5.61)	0.78	
Overall death											
BMI <25 kg/m^2 (n=971)											
n/person-years	651/27121	206/13651		80/7592		22/2352		9/1326			
Multivariable Adjusted	ref	0.77	(0.61-0.98)	0.62	(0.45-0.85)	0.65	(0.37-1.16)	0.38	(0.15-0.96)	<0.001	0.07
Multivariable Adjusted + pre-diagnosis PA	ref	0.83	(0.65-1.06)	0.68	(0.49-0.96)	0.71	(0.40-1.28)	0.41	(0.16-1.05)	0.007	0.07
BMI ≥25 kg/m^2 (n=923)											
n/person-years	708/44070	124/13153		64/6013		19/1296		6/607			
Multivariable Adjusted	ref	0.68	(0.53-0.87)	0.82	(0.57-1.17)	0.92	(0.48-1.78)	0.70	(0.24-2.03)	0.11	
Multivariable Adjusted + pre-diagnosis PA	ref	0.67	(0.52-0.87)	0.81	(0.56-1.18)	0.92	(0.47-1.80)	0.70	(0.24-2.05)	0.16	

Multivariable models adjusted for: estrogen receptor (ER)/progesterone receptor (PR) status, treatment with tamoxifen, aromatase inhibitor, Herceptin, and/or chemotherapy, stage at diagnosis (I-III), pre-diagnosis hormone therapy use, pre-diagnosis BMI, BMI change from pre-diagnosis to current, alcohol consumption, smoking status, aspirin use, and neighborhood SES.

Physical activity reported at least 12 months following diagnosis and at least 24 months prior to the end of follow up, as a cumulative average.

† p value for heterogeneity tests model with BMI (high v. low) in place of continuous BMI vs. interaction model

Note: 3-9 MET-hours/week corresponds to an activity level comparable to approximately 1-3 hours/week of walking at pace of 2.5 mph

Supplementary Table 5. Associations between post-diagnosis strength training (arm and leg weightlifting) activity and breast cancer survival among postmenopausal women: NHS (2000-2016) and NHSII (2001-2017)

	MET-hours of physical activity, per week					
	0	0- <1.0		≥1.0		
	HR (95% CI)	HR (95% CI)		HR (95% CI)		p_{trend}
Breast cancer-specific death (n=166)						
n/person-years	115/17800	33/9336		18/4615		
Multivariable Adjusted	1.0 (ref)	0.55	(0.28-1.12)	0.46	(0.19-1.12)	0.08
Multivariable Adjusted + pre-diagnosis PA	1.0 (ref)	0.50	(0.24-1.06)	0.37	(0.13-1.03)	0.05
Overall death (n=454)						
n/person-years	301/17800	113/9336		40/4615		
Multivariable Adjusted	1.0 (ref)	0.87	(0.60-1.25)	0.62	(0.37-1.05)	0.07
Multivariable Adjusted + pre-diagnosis PA	1.0 (ref)	0.87	(0.60-1.26)	0.65	(0.37-1.12)	0.12

Multivariable models adjusted for: estrogen receptor (ER)/progesterone receptor (PR) status, treatment with tamoxifen, aromatase inhibitor, Herceptin, and/or chemotherapy, stage at diagnosis (I-III), pre-diagnosis hormone therapy use, pre-diagnosis BMI, BMI change from pre-diagnosis to current, alcohol consumption, smoking status, aspirin use, and neighborhood SES.

Physical activity reported at least 12 months following diagnosis with cumulative average of reported activity over follow-up.

Supplementary Table 6. Associations between post-diagnosis strength training (arm and leg weightlifting) activity and breast cancer survival by BMI (updated over follow-up): NHS (2000-2016) and NHSII (2001-2017)

	MET-hours of physical activity, per week						
	0	0- <1.0		≥1.0			
	HR (95% CI)	HR (95% CI)		HR (95% CI)		p_{trend}	p_{het}[†]
Overall death							
BMI <25 kg/m^2 (n=150)							
n/person-years	93/4656	42/3318		15/2049			
Multivariable Adjusted	1.0 (ref)	1.49	(0.42-5.26)	0.39	(0.09-1.76)	0.15	0.79
Multivariable Adjusted + pre-diagnosis PA	1.0 (ref)	1.39	(0.33-5.84)	0.31	(0.06-1.65)	0.11	0.86
BMI ≥25 kg/m^2 (n=190)							
n/person-years	129/8157	42/4161		19/1987			
Multivariable Adjusted	1.0 (ref)	0.51	(0.22-1.21)	0.43	(0.15-1.25)	0.10	
Multivariable Adjusted + pre-diagnosis PA	1.0 (ref)	0.57	(0.23-1.39)	0.41	(0.13-1.34)	0.13	

Multivariable models adjusted for: estrogen receptor (ER)/progesterone receptor (PR) status, treatment with tamoxifen, aromatase inhibitor, Herceptin, and/or chemotherapy, stage at diagnosis (I-III), pre-diagnosis hormone therapy use, pre-diagnosis BMI, BMI change from pre-diagnosis to current, alcohol consumption, smoking status, aspirin use, and neighborhood SES. Physical activity reported at least 12 months following diagnosis with cumulative average of reported activity over follow-up.

Note: Breast cancer-specific survival not included in BMI stratified analyses given n<100 total events in BMI <25 kg/m^2.

[†] p value for heterogeneity tests model with BMI (high v. low) in place of continuous BMI vs. interaction model

Note: 3-9 MET-hours/week corresponds to an activity level comparable to approximately 1-3 hours/week of walking at pace of 2.5 mph

Supplementary Table 7. Individual associations between moderate/vigorous, strength training, and other physical activity and breast cancer survival: NHS (2000-2016) and NHSII (2001-2017)

	HR (95% CI)	HR (95% CI)		HR (95% CI)		p_{trend}
Breast-cancer specific death (n=267)						
Moderate vigorous only, MET-hrs/week	<3	3- <18		≥18		
n/person-years	141/19581	63/11821		11/1395		
Multivariable Adjusted	1.0 (ref)	1.13	(0.64-2.00)	2.02	(0.62-6.59)	0.26
Multivariable Adjusted + pre-diagnosis PA	1.0 (ref)	0.90	(0.48-1.68)	1.73	(0.51-5.94)	0.54
Strength training, MET-hrs/week	0	0-1		≥1		
n/person-years	140/17020	50/10611		25/5167		
Multivariable Adjusted	1.0 (ref)	0.67	(0.36-1.25)	0.74	(0.34-1.61)	0.42
Multivariable Adjusted + pre-diagnosis PA	1.0 (ref)	0.66	(0.35-1.23)	0.63	(0.28-1.40)	0.23
Other physical activity, MET-hrs/week	<3	3- <18		≥18		
n/person-years	59/5611	109/17244		47/9944		
Multivariable Adjusted	1.0 (ref)	0.63	(0.32-1.25)	0.32	(0.14-0.75)	0.01
Multivariable Adjusted + pre-diagnosis PA	1.0 (ref)	0.62	(0.28-1.37)	0.23	(0.09-0.63)	0.001
Overall death (n=732)						
Moderate vigorous only, MET-hrs/week	<3	3- <18		≥18		
n/person-years	375/19581	128/11821		15/1395		
Multivariable Adjusted	1.0 (ref)	0.83	(0.59-1.18)	1.39	(0.62-3.10)	0.88
Multivariable Adjusted + pre-diagnosis PA	1.0 (ref)	0.75	(0.52-1.08)	1.12	(0.48-2.61)	0.62
Strength training, MET-hrs/week	0	0-1		≥1		
n/person-years	332/17020	137/10611		49/5167		
Multivariable Adjusted	1.0 (ref)	1.03	(0.72-1.46)	0.88	(0.54-1.45)	0.58
Multivariable Adjusted + pre-diagnosis PA	1.0 (ref)	0.98	(0.69-1.40)	0.82	(0.49-1.36)	0.40
Other physical activity, MET-hrs/week	<3	3- <18		≥18		
n/person-years	141/5611	271/17244		106/9944		
Multivariable Adjusted	1.0 (ref)	0.86	(0.60-1.25)	0.45	(0.28-0.72)	<0.001
Multivariable Adjusted + pre-diagnosis PA	1.0 (ref)	0.86	(0.56-1.33)	0.36	(0.20-0.63)	<0.001

Multivariable models adjusted for: estrogen receptor (ER)/progesterone receptor (PR) status, treatment with tamoxifen, aromatase inhibitor, Herceptin, and/or chemotherapy, stage at diagnosis (I-III), pre-diagnosis hormone therapy use, pre-diagnosis BMI, BMI change from pre-diagnosis to current, alcohol consumption, smoking status, aspirin use, and neighborhood SES, plus additionally adjusted for other types of physical activity in the tables. Physical activity reported at least 12 months following diagnosis with cumulative average of reported activity over follow-up.

Note: 3-9 MET-hours/week corresponds to an activity level comparable to approximately 1-3 hours/week of walking at pace of 2.5 mph

Supplementary Table 8. Individual associations between moderate/vigorous and other physical activity and breast cancer survival: NHS (1986-2016) and NHSII (1989-2017)

	HR (95% CI)	HR (95% CI)		HR (95% CI)		p_{trend}
Breast-cancer specific death (n=858)						
Moderate vigorous only, MET-hrs/week	<3	3- <18		≥18		
n/person-years	578/77939	244/43709		34/6515		
Multivariable Adjusted	1.0 (ref)	0.90	(0.74-1.10)	0.75	(0.47-1.19)	0.25
Multivariable Adjusted + pre-diagnosis PA	1.0 (ref)	0.87	(0.71-1.07)	0.71	(0.44-1.14)	0.18
Other physical activity, MET-hrs/week	<3	3- <18		≥18		
n/person-years	165/18902	517/79035		174/30203		
Multivariable Adjusted	1.0 (ref)	0.86	(0.68-1.08)	0.79	(0.60-1.05)	0.15
Multivariable Adjusted + pre-diagnosis PA	1.0 (ref)	0.81	(0.63-1.04)	0.72	(0.52-0.99)	0.09
Overall death (n=1,748)						
Moderate vigorous only, MET-hrs/week	<3	3- <18		≥18		
n/person-years	1270/77939	425/43709		51/6515		
Multivariable Adjusted	1.0 (ref)	0.76	(0.66-0.87)	0.68	(0.48-0.95)	<0.001
Multivariable Adjusted + pre-diagnosis PA	1.0 (ref)	0.75	(0.65-0.86)	0.67	(0.47-0.94)	<0.001
Other physical activity, MET-hrs/week	<3	3- <18		≥18		
n/person-years	332/18902	1076/79035		338/30203		
Multivariable Adjusted	1.0 (ref)	0.83	(0.72-0.97)	0.72	(0.60-0.87)	<0.001
Multivariable Adjusted + pre-diagnosis PA	1.0 (ref)	0.83	(0.70-0.98)	0.69	(0.56-0.86)	0.001

Multivariable models adjusted for: estrogen receptor (ER)/progesterone receptor (PR) status, HER2 status, treatment with tamoxifen, aromatase inhibitor, Herceptin, and/or chemotherapy, stage at diagnosis (I-III), pre-diagnosis hormone therapy use, pre-diagnosis BMI, BMI change from pre-diagnosis to current, alcohol consumption, smoking status, aspirin use, and neighborhood SES, plus additionally adjusted for other type of physical activity in the tables. Physical activity reported at least 12 months following diagnosis with cumulative average of reported activity over follow-up.

Note: 3-9 MET-hours/week corresponds to an activity level comparable to approximately 1-3 hours/week of walking at pace of 2.5 mph

Supplementary Table 9. Associations between post-diagnosis overall and moderate/vigorous physical activity and survival following a breast cancer diagnosis with 2-year lagged update of exposure: NHS (1986-2016) and NHSII (1989-2017)

	MET-hours of physical activity, per week									
	<3	3- <9		9 - <18		18 - <27		≥27		
	HR (95% CI)	HR (95% CI)		HR (95% CI)		HR (95% CI)		HR (95% CI)		p_{trend}
Total physical activity										
Breast cancer-specific death (n=1,025)										
n/person-years	129/11005	292/31584		297/37785		127/16861		179/23219		
Multivariable Adjusted	ref	0.86	(0.65-1.14)	0.82	(0.62-1.08)	0.81	(0.58-1.13)	0.78	(0.57-1.07)	0.20
Multivariable Adjusted + pre-diagnosis PA	ref	0.84	(0.62-1.13)	0.78	(0.56-1.07)	0.77	(0.52-1.13)	0.75	(0.51-1.09)	0.27
Overall death (n=2,367)										
n/person-years	317/11005	704/31584		704/37785		294/16861		347/23219		
Multivariable Adjusted	ref	0.78	(0.66-0.93)	0.71	(0.60-0.84)	0.67	(0.55-0.83)	0.56	(0.46-0.68)	<0.001
Multivariable Adjusted + pre-diagnosis PA	ref	0.79	(0.65-0.94)	0.71	(0.61-0.87)	0.67	(0.52-0.84)	0.55	(0.43-0.70)	<0.001
Moderate/vigorous physical activity										
Breast cancer-specific death (n=1,020)										
n/person-years	680/74014	192/26564		98/14681		29/3280		20/2370		
Multivariable Adjusted	ref	0.95	(0.77-1.17)	0.90	(0.69-1.18)	0.91	(0.54-1.52)	0.65	(0.35-1.21)	0.15
Multivariable Adjusted + pre-diagnosis PA	ref	0.95	(0.77-1.18)	0.91	(0.69-1.21)	0.93	(0.55-1.57)	0.66	(0.35-1.25)	0.21
Overall death (n=2,348)										
n/person-years	1673/74014	407/26564		195/14681		44/3280		28/2370		
Multivariable Adjusted	ref	0.78	(0.68-0.90)	0.76	(0.63-0.91)	0.73	(0.50-1.07)	0.64	(0.40-1.03)	<0.001
Multivariable Adjusted + pre-diagnosis PA	ref	0.81	(0.70-0.93)	0.80	(0.66-0.97)	0.79	(0.54-1.17)	0.70	(0.44-1.13)	0.01

Multivariable models adjusted for: estrogen receptor (ER)/progesterone receptor (PR) status, treatment with tamoxifen, aromatase inhibitor, Herceptin, and/or chemotherapy, stage at diagnosis (I-III), pre-diagnosis hormone therapy use, pre-diagnosis BMI, weight change from pre-diagnosis to current, alcohol consumption, smoking status, aspirin use, and neighborhood SES. Physical activity reported at least 12 months following diagnosis as a cumulative average with lagged update of physical activity exposure.

Note: 3-9 MET-hours/week corresponds to an activity level comparable to approximately 1-3 hours/week of walking at pace of 2.5 mph

Supplementary Table 10. Association between change in physical activity from pre- to post-diagnosis and survival following a breast cancer diagnosis: NHS (1986-2016) and NHSII (1989-2017). Follow-up for mortality outcomes begins at 4 years post-diagnosis.

	Increase or decrease in MET-hours per week from pre- to post-diagnosis													
	Decrease 18+		Decrease 9-<18		Decrease 3-<9		Stable	Increase 3-<9		Increase 9-<18		Increase ≥18		
	HR (95% CI)		HR (95% CI)		HR (95% CI)			HR (95% CI)		HR (95% CI)		HR (95% CI)		p_{trend}
Breast cancer-specific death (n=744)														
n/person-years	54/2671		61/3489		82/5118		328/17353	94/5532		59/4064		66/5322		
Multivariable Adjusted	0.97	(0.64-1.47)	0.81	(0.56-1.18)	1.04	(0.76-1.43)	ref	0.78	(0.58-1.05)	0.82	(0.57-1.17)	0.82	(0.59-1.14)	0.29
Multivariable Adjusted + pre-diagnosis PA	1.05	(0.65-1.70)	0.84	(0.57-1.23)	1.05	(0.77-1.44)	ref	0.78	(0.58-1.05)	0.82	(0.57-1.18)	0.83	(0.59-1.16)	0.16
Overall death (n=2256)														
n/person-years	139/2586		217/3333		289/4911		988/16693	242/5384		174/3949		207/5181		
Multivariable Adjusted	0.90	(0.70-1.14)	0.88	(0.73-1.08)	0.88	(0.74-1.04)	ref	0.75	(0.63-0.90)	0.78	(0.64-0.97)	0.77	(0.64-0.93)	0.01
Multivariable Adjusted + pre-diagnosis PA	1.12	(0.85-1.48)	0.98	(0.80-1.21)	0.91	(0.77-1.08)	ref	0.76	(0.63-0.91)	0.80	(0.65-0.99)	0.80	(0.66-0.97)	<0.01

Multivariable models adjusted for: estrogen receptor (ER)/progesterone receptor (PR) status, treatment with tamoxifen, aromatase inhibitor, Herceptin, and/or chemotherapy, stage at diagnosis (I-III), pre-diagnosis hormone therapy use, pre-diagnosis BMI, weight change from pre-diagnosis to current, alcohol consumption, smoking status, aspirin use, and neighborhood SES. Post-diagnosis physical activity reported at least 12 months following diagnosis. Note additional deaths included here as exclusions were not made when physical activity was missing.

Note: 3-9 MET-hours/week corresponds to an activity level comparable to approximately 1-3 hours/week of walking at pace of 2.5 mph
